# Neuroinflammation as the Underlying Mechanism of Postoperative Cognitive Dysfunction and Therapeutic Strategies

**DOI:** 10.3389/fncel.2022.843069

**Published:** 2022-03-28

**Authors:** Zhichao Li, Youzhuang Zhu, Yihan Kang, Shangyuan Qin, Jun Chai

**Affiliations:** ^1^Department of Anesthesiology, Shengjing Hospital of China Medical University, Shenyang, China; ^2^Department of Anesthesiology, The Affiliated Hospital of Qingdao University, Qingdao, China

**Keywords:** surgical trauma, general anesthesia, peripheral inflammation, neuroinflammation, postoperative cognitive dysfunction, preventive strategies

## Abstract

Postoperative cognitive dysfunction (POCD) is a common neurological complication following surgery and general anesthesia, especially in elderly patients. Severe cases delay patient discharge, affect the patient’s quality of life after surgery, and are heavy burdens to society. In addition, as the population ages, surgery is increasingly used for older patients and those with higher prevalences of complications. This trend presents a huge challenge to the current healthcare system. Although studies on POCD are ongoing, the underlying pathogenesis is still unclear due to conflicting results and lack of evidence. According to existing studies, the occurrence and development of POCD are related to multiple factors. Among them, the pathogenesis of neuroinflammation in POCD has become a focus of research in recent years, and many clinical and preclinical studies have confirmed the correlation between neuroinflammation and POCD. In this article, we reviewed how central nervous system inflammation occurred, and how it could lead to POCD with changes in peripheral circulation and the pathological pathways between peripheral circulation and the central nervous system (CNS). Furthermore, we proposed some potential therapeutic targets, diagnosis and treatment strategies at the cellular and molecular levels, and clinical applications. The goal of this article was to provide a better perspective for understanding the occurrence of POCD, its development, and preventive strategies to help manage these vulnerable geriatric patients.

## Introduction

Postoperative cognitive dysfunction (POCD) is a recognized central nervous system (CNS) complication, especially in the elderly that is related to cognitive function changes in patients after anesthesia for surgery (Monk et al., 2008; Terrando et al., 2011a). The clinical phenomenon is characterized by poor memory, comprehension, and attention in patients after surgery and general anesthesia (Mashour et al., 2015; Gold and Forryan, 2019), which seriously affect the quality of life of patients who undergo surgery. Additionally, their discharge time is prolonged, causing a serious burden to the family and society (Steinmetz et al., 2009; Deo et al., 2011; Hovens et al., 2012). In recent years, a multispecialty working group recommended “perioperative neurocognitive disorders” (PND) as an overarching term for cognitive impairments diagnosed in the preoperative or postoperative period. This was of great significance in the establishment of clinical unified diagnostic criteria and communication between different fields (Evered et al., 2018a,b,c,d,e,f). PND includes cognitive decline diagnosed before operation (described as neurocognitive disorder), any form of acute event (postoperative delirium), and cognitive decline diagnosed up to 30 days (delayed neurocognitive recovery) and up to 12 months after the procedure (postoperative neurocognitive disorder). Because the main observation of most clinical and preclinical trials was cognitive functional changes within 30 days after surgery, we used “POCD” to define “delayed neurocognitive recovery” and “postoperative neurocognitive disorder” in this review to accurately describe the research progress of this field in recent years. The International Study of Post-operative Cognitive Dysfunction (ISPOCD) is currently the largest multi-center study aimed at clarifying the relationship between anesthesia and POCD. The ISPOCD study concluded that the incidence of cognitive impairment was 26% at 1 week postoperatively, 10% at 3 months postoperatively, and 1% at 2 years postoperatively (except for cardiac surgery) (Moller et al., 1998). When cardiopulmonary bypass devices were used during cardiac surgery, the incidence of cognitive impairment was as high as 53% at 1 week postoperatively and 42% at 5 years postoperatively (Newman et al., 2001). Based on these results, researchers now pay more attention to the occurrence of POCD. In addition, with the growth of the global population, the acceleration of aging, and the increasing expansion of surgical operations, the incidence of POCD has also gradually increased (Weiser et al., 2015, 2016). It is imperative to study the pathophysiological mechanisms and therapeutic strategies of POCD. Although existing studies suggested that the level of education of patients, preoperative cognitive function status, duration of anesthesia, and secondary surgery were all key factors for the occurrence of POCD, the specific physiological mechanism of POCD pathogenesis and optimal therapeutic strategies remain unknown (Ramaiah and Lam, 2009; Krenk et al., 2010; Rundshagen, 2014). In addition, a large number of animal experiments and clinical studies have shown that advanced age is the only independent risk factor for the occurrence and development of POCD (Corona et al., 2012). At present, there are several hypotheses about the pathogenesis of POCD related to central nervous inflammation, cholinergic nervous system dysfunction, nerve cell apoptosis, and oxidative stress injury. Also important are genetic risk factors and the potential role of epigenetic mechanisms in the underlying effects of POCD. Recent studies have shown that the changes at the gene level induced by surgery and anesthesia played an important role in the pathogenesis of POCD (He and Wang, 2021; Schenning et al., 2021). Moreover, emerging evidence has reported that epigenetic regulation was vital for postoperative cognitive function (Chen et al., 2017; Min et al., 2021), and Schenning et al. (2019) have established that the role of sex and genetic variables were vital for POCD in a longitudinal cohort analysis (Schenning et al., 2019). Furthermore, recent research suggested that Cerebral microvascular endothelial glycocalyx (CeGC) is crucial in protecting fragile parenchymal tissue and effective functioning of the BBB, as one particularly important CeGC function is to act as a protective barrier and permeability modulator. CeGC degradation is one of the factors which can lead to an increase in BBB permeability. It occurs naturally in aging, nevertheless, premature degradation has been confirmed in multiple conditions associated with cognitive impairment, such as inflammation, brain edema, cerebral malaria, Alzheimer’s and recently COVID-19 (Stoddart et al., 2022). However, because the most studies have focused on the structure and function of glycocalyx and endothelia as well as the effect on vascular barrier, there was no sufficient evidence to establish the potential correlation between glycocalyx, endothelia, neuroinflammation and POCD. However, a growing number of studies have shown that the neuroinflammatory response still played an initial and central role in the occurrence and development of POCD (Duan et al., 2018; Skvarc et al., 2018; Berger et al., 2019; Lin F. et al., 2021). In this article, the neuroinflammatory mechanisms, prevention, and treatment strategies for POCD are reviewed.

## Potential Mechanisms of Central Neuroinflammation Occurrence

### Peripheral Inflammatory Response Induced by Surgery

The role of anesthesia in the occurrence and development of POCD is still unclear. Previously, many researchers believed that there was no clear correlation between the occurrence of POCD and the choice of different anesthesia regimens and anesthetics (Rasmussen et al., 2003; Bryson and Wyand, 2006; Royse, 2007; Mason et al., 2010; Evered et al., 2011). However, many studies have shown that inhaled anesthetics could cause neurotoxicity in the form of amyloid beta deposition (Xie et al., 2008; Zhang B. et al., 2013). In one study, it was found that the intravenous anesthetic, propofol, caused neuronal cell death in the developing rat brain (Pesić et al., 2009; Chen et al., 2016). And both of these methods impaired postoperative cognitive function (Cai et al., 2012; Geng et al., 2017). Animal experiments have also proven that the occurrence and development of POCD were related to the choice of anesthetics, such as the intravenous anesthetics etomidate and propofol, and the inhaled anesthetic, sevoflurane, all of which have become common induction drugs for constructing animal models of POCD in the field of basic research. Increased attention has been focused on specific induction mechanisms, such as early neuroinflammation, nerve cell death, and sevoflurane-induced cross dysfunction of iron and sugar metabolism in mouse brain cells, which led to apoptosis, along with the corresponding diagnosis and treatment strategies (Cui et al., 2018; Li D. et al., 2020; Ge et al., 2021; Liu et al., 2021). The results of various studies on the effects of different anesthesia methods and anesthetics on postoperative cognitive function have been different, even contradictory, and the reasons for the conflicting results are still inconclusive. After reviewing many studies, we found that there were substantial differences in the methodologies used in the relevant clinical studies on the effects of anesthesia methods and anesthetics on postoperative cognitive outcomes, neurocognitive combination of tests, the test time interval, analysis end points, statistical methods, and how to define the neuropsychological defects. The method used to assess postoperative cognitive function may be the most important factor. For example, Egawa et al. (2016) applied neurocognitive assessment methods such as the Mini-Mental State Examination (MMSE), the trail making test (parts A and B), the number span (forward and backward), and the groove nail board test (dominant and non-dominant hand) in clinical studies to observe if the anesthetic affected postoperative cognitive function in patients with one-lung ventilation (OLV). This prospective study compared the incidence of POCD and intraoperative cerebral oxygen desaturation in OLV patients anesthetized with propofol versus sevoflurane during lung surgery and it was found that there was no statistical difference in the incidence of POCD in both groups (Egawa et al., 2016). However, a large sample study conducted by Zhang et al. (2018) used a more rigorous neuropsychological test process by conducting seven tests on nine subscales (Zhang et al., 2018), and finally concluded that general anesthesia with propofol reduced the incidence of delayed neurocognitive function recovery in elderly patients at 1 week after major cancer surgery compared to general anesthesia with sevoflurane. Although there were differences in the surgical method selection and the inclusion criteria of subjects in these two clinical studies, along with the influence of surgical method difference on the final outcome of the study, it is important to clarify and emphasize the influence of the neurocognitive assessment method on experimental results. The MMSE and the Montreal assessment method are less sensitive and specific than other more complex neurocognitive test combinations, but they are still widely used in clinical studies due to their relative simplicity and efficacious clinical implementation. Considering all these points, we hypothesized the independent contribution of anesthesia to postoperative cognitive outcomes played a much smaller role in the occurrence and development of POCD than the choice of surgical procedures and the extent of the trauma. And it has been reported that in animal experiments (Wan et al., 2007), there was no difference between anesthetized and non-anesthetized rats without surgery. Only in splenectomy rats was the activation of glial cells and increase of pro-inflammatory cytokines in the hippocampus observed and resulted in cognitive impairment. Furthermore, a clinical study (Baigrie et al., 1992) found that the incidence of POCD after major heart and abdominal surgery was higher than that of hernia repair and limb surgery because major surgery induced greater trauma and increased the levels of relevant inflammatory factors released in the body (Lundin et al., 2020). Another study conducted by Yu et al. (2017) confirmed the potential impact of surgical trauma and surgical complexity on the degree of inflammatory response and the incidence of POCD in a prospective cohort study. Additionally, a randomized, allocation-concealed, open-label, multicenter clinical trial recently showed that in patients aged 65 years and older undergoing hip fracture surgery, regional anesthesia without sedation did not significantly reduce the incidence of postoperative delirium compared with general anesthesia (Li et al., 2021). This helped to prove that the choice of anesthesia method did not have a statistically significant effect on brain function, and it was the stress of the type of surgery that played a more important role in cognitive impairment.

Surgical stress causes inflammation and immune activation, which lead to a local inflammatory response and a systematic cascade of inflammatory signaling molecules. However, many studies have shown that immune activation could trigger POCD and could also induce, maintain, and aggravate neuroinflammation. In general, however, the immune activation in response to cognitive impairment was usually chronic, whereas the effects of transient and more acute immune activation inside and outside of the brain were more difficult to predict, qualitatively or quantitatively. Perioperative neuroinflammation may play a key role in the development of POCD, and other potential factors that may contribute include accelerated aging of neurons, neuroendocrine disorders, and circadian rhythm disorders (Skvarc et al., 2018). This article discussed the potential mechanism of neuroinflammation during the occurrence and development of POCD. Aseptic trauma and local cell necrosis caused by surgery result in the release of damage associated molecular patterns (DAMPs), including high mobility group protein B1 (HMGB1), into the extracellular system. HMGB1 is a highly conserved nuclear protein under normal physiological conditions but is an inflammatory factor and signal of injury under pathological conditions (Cohen et al., 2009; Zhang et al., 2010; Bianchi et al., 2017). When HMGB1 is released from the nucleus into the cell, it passes through pattern recognition receptors (PPRs), such as toll-like receptor (TLR) and receptor for advanced glycation end products (RAGE), to activate related signaling pathways. Furthermore, activated immune cells (peripheral macrophages and monocytes) are recruited to the site of injury, and activation of the intracellular NF-κB pathway promotes the synthesis and release of various inflammatory factors (IL-1β, IL-6, and TNF-α) by peripheral immune cells. Proinflammatory factors promote the secretion of HMGB1 through a positive feedback loop, inducing and maintaining the peripheral inflammatory response (Zhang et al., 2010; Bianchi et al., 2017; Skvarc et al., 2018). Studies have reported that the level of HMGB1 in the serum of POCD patients was significantly higher than that of the control group, which further supported the potential correlation between HMGB1 and POCD (Lin et al., 2014; Yu et al., 2017). Moreover, some studies have found that surgical trauma not only led to the upregulation of peripheral HMGB1 concentration, but also increased expression levels of HMGB1 and its receptors in the hippocampus. The expression of HMGB1 in the hippocampus may be related to the destruction of the integrity of the blood–brain barrier (BBB) (He et al., 2012). The central nervous inflammation hypothesis for POCD posited that changes in peripheral inflammatory factors could affect the CNS and subsequently cause CNS inflammatory responses. Wan et al. (2007) found in animal experiments that increased levels of inflammatory factors (IL-1, IL-6, and TNF-α), could be detected in the CNS after non-craniocerebral surgery and were positively correlated with inflammatory factors in peripheral blood. This suggested that the inflammatory response of the body after non-craniocerebral surgery could potentially affect the CNS and cause CNS inflammation, which further supported the central nervous inflammation hypothesis for POCD. Therefore, the key to understanding the influence of surgical trauma on postoperative cognitive outcome is exploring how peripheral inflammation affected the CNS and how CNS inflammation induces the loss of cognitive function.

### Association Between Peripheral and Central Inflammatory Responses

**FIGURE 1:**
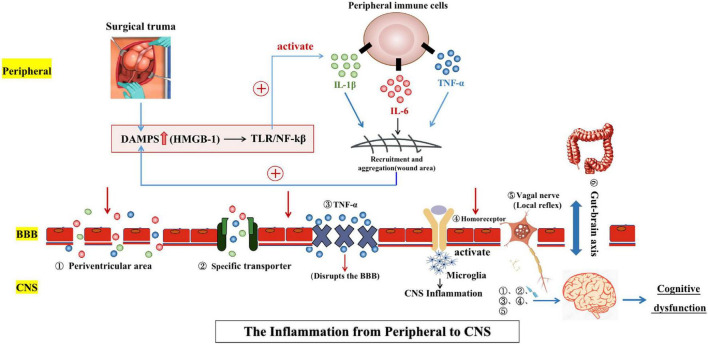
Surgical trauma stimulates the peripheral inflammatory response and immune activation. Injury-related pattern molecules dominated by HMGB1 recruit and activate peripheral immune cells and also promote the synthesis and release of a variety of inflammatory factors. Many inflammatory factors can be positively fed back to the secretion pathway for HMGB1 to induce and maintain the peripheral inflammatory response. Peripheral inflammation can spread to the central nervous system through several pathways [(1) Periventricular area; (2) Specific transporter; (3) TNF-α; (4) Homoreceptor; (5) Vagal nerve; and (6) Gut-brain axis], leading to central neuroinflammation and ultimately cognitive dysfunction. The two-way communication between the gut microbiome and the brain, termed the “gut-brain axis,” is involved in brain function and cognitive regulation.

The systemic inflammatory response caused by surgery increased the plasma levels of IL-1, IL-6, TNF-α, and other inflammatory factors (Peng et al., 2013; Lin et al., 2014; Sun et al., 2014) which induced the inflammatory response in the CNS through various mechanisms (Figure 1). At present, the literature suggested the mechanisms were the following: Inflammatory factors entered the CNS by passive diffusion in the periventricular area along the concentration gradient due to the lack of a continuous BBB and the physiological characteristics of relative permeability. In the intact BBB region, inflammatory factors could also be actively transported into the CNS through specific transporters. In addition, the permeability of the BBB changed under pathological conditions. The release of TNF-α in the perioperative systemic inflammatory response was thought to increase BBB permeability and promote neuroinflammation, delirium, and subsequent POCD (Terrando et al., 2011b; Wen et al., 2020). Moreover, a large number of animal experiments have shown that surgical trauma promoted the release of peripheral TNF-α, thereby damaging the integrity of the BBB (activation of the NF-κB pathway may be the mechanism), leading to the infiltration of a large number of peripheral inflammatory cells (mainly macrophages) and inflammatory factors into the CNS and further inducing inflammatory responses in the CNS (Terrando et al., 2011b; He et al., 2012; Cheon et al., 2017). Furthermore, it was found that inflammatory stimulation followed by treatment with TNF-α antagonists resulted in improved cognitive performance compared to the control group (Yang et al., 2016; Lin S.-Y. et al., 2021). All of the above mechanisms are related to the direct entry of peripheral inflammatory factors into the center to induce the CNS inflammatory response, but the inflammatory response of CNS may also be induced by the interaction of peripheral inflammatory factors through a variety of signal transduction pathways. These mechanisms included the following: Peripheral inflammatory factors (IL-1) directly bound to sites with homologous receptors on the BBB endothelial cells and them to produce immunoactive molecules under the stimulation of peripheral signals, activating central microglia and astrocytes and inducing the immune response of the CNS (Banks et al., 1995). In addition, vagal afferent nerves can rapidly activate central inflammatory pathways and induce CNS inflammatory responses when stimulated by surrounding inflammatory factors. Primary afferent activation initiates local reflexes (cardiovascular and gastrointestinal), which bolster host defense. Immune cells first respond to inflammatory stimuli and release inflammatory factors and other mediators to activate nerve elements, including primary afferent neurons in the vagal sensory ganglion. Vagal sensory neurons can express the mRNAs of IL-1 and prostaglandin receptors, and peripheral inflammatory mediators bind to the corresponding receptors on vagal afferent nerve fibers to activate the immune response in the CNS (Goehler et al., 2000, 2005; Dantzer et al., 2008). The link between gut microbes and the brain was named the “gut-brain axis” (Smith, 2015). The gut microbiota is made up of intestinal flora composed of a series of bacteria, viruses, and other microorganisms. Remarkably, dysregulation of gut microbiota promoted the progression of neurodegenerative diseases and the release of inflammatory markers such as IL-6 and TNF-α (Yang et al., 2018; Dalton et al., 2019). In recent years, increasing numbers of animal experiments have confirmed that surgery and anesthesia could induce intestinal microflora imbalance and then affected brain cognitive function through certain mechanisms (Zhan et al., 2019; Lian et al., 2021). These pathological mechanisms could be ameliorated by the increase of beneficial bacteria in the gut, such as *Bifidobacterium*, *Lactobacillus*, and Galactose oligosaccharide (Jeong et al., 2015; Kobayashi et al., 2017; Yang et al., 2018). Furthermore, the latest research has shown that proper exercise improved postoperative neuroplasticity and cognitive function by ameliorating the gut dysbiosis and valeric acid increase (Lai et al., 2021). However, it is still unclear how the gut microbiota and neurons in the brain mutually interacted and how these interactions affected normal brain cognitive function (Chen et al., 2021).

It should be noted that the CNS traditionally relies on the presence of the BBB and blood-cerebrospinal fluid barrier, which make the brain an independent organ at the immune level and is not affected by the peripheral immune system. However, Iliff et al. (2015) recently discovered the existence of lymphatic pathways in the CNS, and another research team has found that meningeal lymphatic pathways in the CNS communicated with the peripheral immune system (Louveau et al., 2015). These findings helped to disprove the hypothesis of the brain as an immune-privileged organ.

## Relationship Between Central Neuroinflammation and Postoperative Cognitive Dysfunction

Peripheral immune signals are transmitted to the CNS through the above-mentioned neural and humoral pathways to activate central resident immune cells, namely microglia cells. Microglia, as the inherent immune cells of the CNS, have a lineage similar to peripheral blood macrophages, and the activation of microglia cells is a key link in the occurrence and development of the central neuroinflammatory response (Wan et al., 2007; Feng et al., 2017; Safavynia and Goldstein, 2018). Activation of microglia cells can produce a series of cytokines (IL-1β, IL-6, and TNF-α) thus inducing the central inflammatory response. In addition, excessive release of inflammatory cytokines can activate microglia cells in turn, forming a vicious cycle that further aggravates the inflammatory damage of the CNS and impairs cognitive function. In this process, the activation of microglia cells and the specific mechanism by which CNS inflammation causes cognitive impairment are the two central links, which are also the breakthrough points for the clinical diagnosis and treatment of POCD.

The activation mechanism for microglia is believed to be closely related to toll-like receptors (TLRs). Members of the TLR family are expressed on the serosal and endosomal membranes of myeloid cells and play an important role in innate immune responses. The TLR4 pathway provides important mechanisms for microglia to recognize pathogen-associated molecular patterns (PAMPs), including pathogen and endogenous stress injury alarms. TLR4 deficiency downregulated proinflammatory mediators and prevented nerve injury in cerebral ischemia and neurodegenerative diseases (Minichiello et al., 2002; Lu et al., 2015). In recent studies, TLR3 has been reported that plays an important role in memory based on the hippocampal anatomy. Chen C. et al. (2019) found that TLR3 overexpression after surgical injury was an endoreceptor for extracellular RNAs (exRNAs), especially double-stranded RNAs (dsRNAs), through a POCD animal model study. Compared with the sham operation group, the TLR3 expression levels increased in the operation group and colocalized with neurons and microglia. A TLR3 deficiency significantly reduced the central inflammatory response and apoptosis after unilateral nephrectomy to improve hippocampus-dependent memory (Chen C. et al., 2019). In this experiment, cell necrosis and an increase in exRNA levels were induced by freezing and then thawing to simulate the injury caused by surgery. Although this simulation method has been proven to be effective, the correlations between the results *in vitro* and *in vivo* are limited; thus, the experiment cannot fully reflect the actual pathogenesis of POCD in the human body. Furthermore, recent study has also evidenced that TLR2 contributes to surgery-induced neuroinflammation and cognitive impairment, moreover, HMGB1 upregulates TLR2 expression in the hippocampus after surgery to expedite this contribution (Lin F. et al., 2021). Consequently, TLR2 and HMGB1 are underlying targets for improving POCD. As the most important brain region for learning and memory, the changes in the structure and function of the hippocampus directly affect the cognitive ability of patients. Moreover, due to the high expression of various PPRs for inflammatory factors in the hippocampus (TNF-α and IL-1β), when inflammatory factors in the CNS are overexpressed, hippocampal neurons are directly and indirectly damaged. Additionally, the formation of hippocampal neurons and the plasticity of hippocampal morphology related to learning or memory are inhibited, ultimately leading to impaired cognitive function (Sierra et al., 2014). In addition, brain-derived neurotrophic factor (BDNF) is a neurotrophin widely expressed in the CNS that plays a key role in neuronal survival and differentiation as well as synaptic plasticity through activation of TrkB-FL (full-length receptor of tyrosine kinase receptor B) (Lewin and Barde, 1996; Lu et al., 2013). BDNF influences synaptic plasticity by regulating the formation and modification process of proteins on synapses, which makes BDNF a crucial regulator of synaptic transmission and long-term potentiation (LTP) in the hippocampus and other brain regions. Furthermore, BDNF plays a crucial role in specific forms of memory formation mediated by Tyrosine Kinase receptor B (TrkB), and the production of a large number of inflammatory mediators inhibit the BDNF/TrkB signaling pathway, resulting in the synaptic dysfunction involved in the pathogenesis of POCD (Minichiello et al., 2002; Leal et al., 2014; Qiu et al., 2016a,^2020^). In a study using 3-month-old male rats that underwent abdominal surgery as the experimental group and a non-operation group as the control group, the cognitive function, neuroinflammatory markers, and BDNF of the rats were monitored after surgery. Postoperatively, the rats showed changes in exploratory activity, which were associated with increased plasma IL-6 levels. Spatial learning and memory functions were temporarily impaired 2 weeks after surgery, while non-spatial cognition seemed unaffected. Analysis of the brain tissue showed increased neuroinflammation (IL-1β and microglial proliferation) at 1 week postoperatively, decreased BDNF levels at 2 and 3 weeks postoperatively, and decreased neurogenesis until at least 3 weeks following surgery. These findings showed that only spatial learning and memory were affected by surgery in young adult rats, suggesting hippocampus-dependent cognition was especially vulnerable to surgery-induced impairment. It also suggested potential relationships between postoperative neuroinflammation, BDNF levels, and cognitive function (Hovens et al., 2014). Furthermore, a recent study has confirmed that the neuroinflammation overactivated N-methyl-D-aspartate receptors (NMDARs) play a key role in overactivation of calpain, cleavage of TrkB-FL receptor, BDNF/TrkB signaling dysfunction, dendritic spine loss, cell apoptosis, and consequent cognitive impairments (Qiu et al., 2020). Although if signaling is the initial trigger for inflammation to NMDAR overactivation is still unknown, we can identify feasible therapeutic strategies to tackle abnormal activation of NMDARs or calpain that may provide effective interventions for POCD.

The specific physiological mechanisms for the effect of the neuroinflammatory reactions on POCD pathogenesis have not been precisely defined, but a large number of studies have confirmed the negative correlation between the two from various aspects, and accumulating evidence suggested that neuroinflammation played an initial and central role in cognitive impairments after surgery and anesthesia (Cibelli et al., 2010; Qiu et al., 2016a,b). Li D. et al. (2020) used etomidate to establish a mouse model of perioperative cognitive dysfunction and found that microglia activated at the initial pathological stage of POCD triggered the activation of α1-specific astrocytes, thereby inducing long-term synaptic inhibition and cognitive deficits. Neurofilaments (NFs) play an important role in axonal structure and nerve conduction. Abnormal degradation of NFs is often associated with degenerative diseases and is also characterized by excessive neuroinflammation in the brain. Although, it is unclear whether NFs are involved in the occurrence and development of POCD, Trichostatin A (TSA) was used to pretreat the experimental group, and the results showed that TSA reduced neuroinflammation and NF damage, significantly improving postoperative cognition. The Kyoto Encyclopedia of Genes and Genomes (KEGG) database is used to systematically analyze the metabolic pathways and functions of gene products in cells. KEGG enrichment analysis showed that compared to the surgery group, there were nine pathways enriched in the TSA+ surgery group, among which two signaling pathways were closely related to the changes in the NF proteins (Cao et al., 2020). These results enhanced our understanding of the pathogenesis and development of POCD and suggested potential therapeutic targets.

## Some Preventive Strategies for the Neuroinflammation Pathogenesis of Postoperative Cognitive Dysfunction

In recent years, studies have shown that neural inflammation in POCD played a key role in the development process. With this point of view, more attention has been focused on the prevention and treatment of POCD based on the neuroinflammatory response. The research direction included the direct application of anti-inflammatory drugs, inhibition of inflammatory signaling pathways, and regulation of microglia. The latest research progress on the prevention and treatment strategies related to POCD neuroinflammation are reviewed below (Table 1).

**TABLE 1:** From different perspectives, prevention and treatment strategies were adopted for the neuroinflammation pathogenesis of POCD.

Study focus			

	**Basis and principle**	**Specific strategies**	**References**
M1-M2 phenotypic transformation	M1↑ M2 ↓	★ Cerium oxide nanoparticles	Zeng et al., 2018
		★ Resveratrol	Yang et al., 2017
		★ GSK-3β inhibitors	Li J. et al., 2020
		★ Erythropoietin	Lee et al., 2017
Anti-inflammatory drugs	Inflammatory response Anti-inflammatory activity	★ COX-2 inhibitors	Xu et al., 2014; Huang et al., 2020
		★ Dexmedetomidine	Liu H. et al., 2017; Wang et al., 2018; Xu et al., 2018
		★ TNF-α receptor antagonist	Yang et al., 2016
		★ IL-1 receptor antagonist	Barrientos et al., 2012
		★ HMGB1 antibody	Terrando et al., 2016
		★ Cholinesterase inhibitors	Spies et al., 2021
		★α7nAchR agonist	Liu P.-R. et al., 2017; Alzarea and Rahman, 2019; Gong et al., 2020
Some other recent arguments at the molecular level	Acetylation of tau TIR→NADase→neurodegeneration Regulation of microglia CAP ↑ NMDAR/Ca^2+^/Calpain↓ CX3CR1/L1 signaling↓	★ Resveratrol →IRT1 expression	Yan et al., 2020
		★ Silencing SARM1	Jiang et al., 2020; Lin H. et al., 2021
		★ MicroRNA-124 expression	Chen Y. et al., 2019
		★ Silencing SP1 →α7nAchR agonist	Lv et al., 2020
		★ Memantine or MDL-28170	Qiu et al., 2020
		★ Antibody of blockade	Cho et al., 2021

*TIR, toll/IL-1 receptor domain. Toll-like receptors from different sources all have the TIR with highly conserved sequence, most of which are located at the c-terminal of Toll receptor (Only a few TIR of plant disease-resistant proteins are located at the N-terminal), located in the cell, and play an important role in signal transduction of natural immunity. CAP, cholinergic anti-inflammatory pathway. The cholinergic anti-inflammatory pathway is a neural mechanism that inhibits pro-inflammatory cytokine release via signals that require the vagus nerve and α7 receptors.*

### Neuroprotective Effects Can Be Achieved by Regulating Phenotypic Polarization of Microglia

As the resident immune cells in the brain, microglia cells are the main immune line of defense in the CNS. Microglia are in the resting state (M0 phenotype) under physiological conditions and play a role in immune surveillance. However, in the pathological state, microglia are rapidly activated with transcriptional adaptive function changes and eventually transition to the classical activated type (M1 phenotype). M1 microglia kill pathogens by releasing proinflammatory factors and toxic substances, while alternatively activated microglia (M2 phenotype) achieve neuroprotective effects by promoting tissue repair and regeneration. Clinical studies have shown that overactivated microglia of the M1 phenotype can cause neuronal incapacity injury and degeneration and play an important role in cerebrovascular diseases, neurodegenerative diseases, neurodevelopmental disorders, and psychiatric diseases (Alvarez et al., 2016; Xiong X.-Y. et al., 2016). Therefore, regulation of the phenotypic polarization of microglia provided a potential new strategy for the treatment of some psycho-neurological diseases.

Inhibiting the M1 phenotype while stimulating the M2 phenotype is considered a potential therapeutic approach for the treatment of neuroinflammation-related diseases. Zeng et al. (2018) found that cerium oxide nanoparticles scavenged a variety of reactive oxygen species with high efficiency and drove microglia polarization from the proinflammatory phenotype (M1) to the anti-inflammatory phenotype (M2) under pathological conditions. The pretreatment of these nanoparticles changed the function of microglia by blocking proinflammatory signals, and the effect on neuronal cells was transformed from damage to protection. Although the clinical practicability of cerium oxide application in the prevention and treatment of POCD still needs to be verified, this discovery was significant in the field of applied chemistry and provides a direction for POCD interdisciplinary research and interdisciplinary cooperation. Yang et al. (2017) established a mouse model of central nervous inflammation induced by lipopolysaccharides and found that resveratrol reduced inflammatory damage and promoted microglial cell polarization to the M2 phenotype. The mechanism was related to resveratrol overexpression of peroxisome proliferator-activated receptor-γ coactivator-1α (PGC-1α), which can improve the behavior of lipopolysaccharide-induced disease in mice. Glycogen synthetic kinase 3β (GSK-3β) is a widely expressed kinase that plays different roles in different cell types, and its role in regulating innate immune activation has been well documented, although its specific mechanism in POCD is still unclear. However, recent studies have shown that GSK-3β played a key role in microglia polarization. Experimental treatment with the GSK-3β inhibitor, lithium chloride (LiCl), induced microglia transformation from M1 to M2 during neuroinflammation (it should be noted that LiCl alone has no effect on microglia polarization), thus improving recognition. This protein may be an important target for the treatment of POCD in elderly patients (Li J. et al., 2020).

Erythropoietin (EPO) is a hematopoietic hormone that plays an anti-inflammatory role by affecting macrophage function. The cognate receptor for EPO is expressed in various cell types; thus, EPO has pleiotropic functions in non-hematopoietic as well as hematopoietic cells (Lombardero et al., 2011). Immune cells also express the EPO receptor and are thus subject to modulation by EPO (Brines and Cerami, 2012). To evaluate the effects of EPO on POCD and macrophage polarization after abdominal surgery, Lee et al. (2017) injected Institute of Cancer Research (ICR) mice with EPO (5000 U/kg) before and after abdominal surgery, with or without the addition of an argininase inhibitor (amino-6-boronic acid, 10 mg/kg) (Lee et al., 2017). Forty-eight hours post-surgery, the researchers assessed memory, synapse function, and macrophage/microglial phenotypes in the spleen and hippocampus and found that EPO inhibited the expression of M1-related genes in the spleen and hippocampus and promoted the expression of the M2 gene. In addition, EPO reduced the ratio of macrophages/microglia expressing the M1 surface marker (CD40) and increased the ratio of macrophages/microglia expressing the M2 surface marker (CD206). This ultimately reduced the production of pro-inflammatory cytokines in the hippocampus, alleviated the synaptic dysfunction related to surgery, and prevented the occurrence and development of POCD. In the present study, to show the beneficial effect of M2 macrophage activation on postoperative cognitive function, we conducted an additional study using a selective argininase inhibitor preoperatively in conjunction with rhEPO. Argininase activity mediated most of the anti-inflammatory effects associated with M2 (Pesce et al., 2009) and was positively correlated with M2 macrophages (Yang and Ming, 2014). We also found that the argininase inhibitor in this study eliminated the beneficial effects of EPO on POCD. Overall, EPO prevented POCD by promoting the transformation of the macrophage phenotype to the M2 type.

### Direct Application of Anti-inflammatory Drugs

A large number of preclinical and clinical trials have shown that non-steroidal anti-inflammatory drugs, anti-TNF-α antibodies, IL-1 receptor antagonists, HMGB1 antibodies, cholinesterase inhibitors, cholinergic agonists, central α7 nicotinic acetylcholine receptor (α7nAchR) agonists, and dexmedetomidine (DEX) significantly reduced CNS inflammation and inflammatory factors in the hippocampus to improve the cognitive function of experimental animals after surgery (Barrientos et al., 2012; Wang et al., 2012, 2018; Xu et al., 2014; Terrando et al., 2016; Yang et al., 2016; Zhu Y.-Z. et al., 2016; Alzarea and Rahman, 2019; Spies et al., 2021). The anti-inflammatory pathways can be divided into the direct reduction of inflammation and indirect reverse enhancement of the anti-inflammatory activity of the cholinergic system. Nonetheless, both pathways negatively regulate inflammation. Research on the application of cyclooxygenase-2 (COX-2) inhibitors has a long history, and many experiments and meta-analyses have confirmed their clinical feasibility as well as safety and efficacy (Huang et al., 2020). As COX-2 inhibitors, ibuprofen, celecoxib, and parecoxib can significantly inhibit the expression of IL-1β, TNF-α, and S100β, reduce the incidence of postoperative POCD, and improve the cognitive function of patients (Xu et al., 2014; Lu et al., 2017; Huang et al., 2020). In addition, DEX has been the focus of clinical POCD prevention in recent years and reduced surgical stress injury and inhibited central nervous inflammation, thus improving postoperative cognitive function. As a highly selective α 2-adrenergic receptor agonist, DEX can provide good sedation and analgesia for patients undergoing surgery but has no obvious inhibitory effects on respiration. According to existing experimental evidence, the anti-inflammatory properties of DEX in animal models have been fully confirmed. The mechanism was complex and included the following processes: Inhibition of the expression of inflammatory cytokines via the nuclear factor-κB pathway, which is a key regulator of the inflammatory cascade and TNF-α activation (Wang L. et al., 2017). DEX alleviated hippocampal injury after abdominal surgery by upregulating the anti-apoptotic protein Bcl-2 and downregulating pro-apoptotic factors Fas caspase-8 and caspase-9 (Xiong B. et al., 2016). Maintenance of normal vagus nerve function was essential for the anti-inflammatory effects of DEX. It was found that DEX did not exert its anti-inflammatory effects in the CNS when the vagus nerve was severed in the animal model of tibial fracture after surgery. The cholinergic anti-inflammatory pathway relied on the preservation of vagus nerve tone, and its normal function was crucial for the beneficial regulation of DEX in neuroinflammation (Zhu Y.-J. et al., 2016). DEX’s enhancement of the cholinergic anti-inflammatory pathway via the α7nAchR mechanisms has also been reported elsewhere (Xu et al., 2018). DEX has been shown to inhibit microglia-mediated release of TNF-α, nitric oxide, interleukin 1β, monocyte chemo attractor protein-1 (McP-1), prostaglandin E2, and other proinflammatory cascade factors, thereby inhibiting CNS inflammation (Peng et al., 2013; Zhang et al., 2015; Chen and Qian, 2016; Liu H. et al., 2017). Interestingly, unlike animal models, the effectiveness of DEX in the prevention and treatment of POCD has not been established in clinical trials, and meta-analyses of different clinical studies have often yielded different conclusions (Man et al., 2015; Zhou et al., 2016; Deiner et al., 2017). The differences between animal models and clinical trials may be due to the limitations of the clinical studies themselves. The differences in diagnostic criteria and neurocognitive endpoints of clinical trials for POCD may be the key to the successful exploration of the effectiveness of DEX in the clinical prevention and treatment of POCD. However, it should be noted that except for COX-2 inhibitors, most of the other neuroinflammatory anti-inflammatory drugs are still in the animal model stage, and we cannot guarantee their safety for human use. In addition, as a self-protection mechanism against injury, the inflammatory response has a dual nature. The clinical safe dose of anti-inflammatory drugs is unknown, and an improper dose is likely to affect the normal physiological function of the body. Therefore, the application of anti-inflammatory drugs in the clinical prevention and treatment of POCD requires further research and demonstration.

### Recent Research Developments on Cellular and Molecular Levels

#### Role of SIRT1 on Postoperative Cognitive Dysfunction

Silencing regulatory protein 1 (SIRT1), a member of the Sirtuin protein family, is a protein deacetylase responsible for cell regulation. SIRT1 is a nicotinamide adenine dinucleotide (NAD)-dependent enzyme that uses NAD substrates to remove acetyl groups from proteins. A study (Yan et al., 2020) showed that SIRT1-deletion-mediated acetylation of Tau (a microtubule-associated protein in nerve cells) led to the hyperphosphorylation of Tau protein in the hippocampus of elderly POCD models, leading to cognitive impairment. Interestingly, in a behavioral test, resveratrol preconditioning almost restored SIRT1 expression, reduced the levels of acetylated Tau and high Tau phosphorylation in the hippocampus, and improved cognitive performance. More importantly, it was observed that microglia-derived neuroinflammation induced by the inhibition of microglia by SIRT1 exacerbated Tau acetylation in cultured neurons *in vitro*. Experimental results supported the idea that SIRT1 activation had dual benefits in the elderly POCD model, which provided an optimistic prospect for the prevention and treatment of POCD in clinical practice. In addition, a study (Shi et al., 2020) found that SIRT1 improved POCD induced by cardiac surgery. SIRT1 expression was significantly inhibited in POCD mouse models after cardiac surgery while activation of SIRT1 via SRT1720 (an agonist for SIRT1) significantly reduced systemic inflammatory factors and restored cognitive function in POCD mice.

#### Role of SARM1 on Postoperative Cognitive Dysfunction

Sterile alpha and toll/interleukin-1 receptor motif-containing protein 1 (SARM1) is a core protein that regulates the process of neurodegeneration. Its toll-interleukin-1 receptor (TIR) domain plays a role in promoting neurodegeneration through NADase activity, triggering axonal degeneration, and destroying neural circuits (Jiang et al., 2020). Lin H. et al. (2021) found through animal experiments that SARM1 was a pathogenic factor for neuroinflammation and cognitive impairment in anesthetically induced elderly mice. They also demonstrated that cognitive impairment in isoflurane anesthetically induced mice could be prevented by the absence of SARM1, making SARM1 an effective therapeutic target for the treatment or prevention of POCD (Lin H. et al., 2021).

#### Role of miR-124/VAMP3 on Postoperative Cognitive Dysfunction

miR-124 is one of the most abundant microRNAs in the brain that regulates the function of microglia. The downstream targets of miR-124 were studied using bioinformatics screening and dual luciferase reporter gene detection verification, and vesicle-associated membrane protein 3 (VAMP3) was identified as a potential target. In a rat model of surgical trauma treated with exogenous miR-124 and electroacupuncture (we found that electroacupuncture specifically increased miR-124 expression in the hypothalamus and hippocampus), Chen Y. et al. (2019) found that increased miR-124 expression was accompanied by decreased VAMP3 expression and resulted in decreased release of inflammatory cytokines associated with microglial activation after surgery. Our study showed that miR-124/VAMP3 was involved in surgical-induced microglial activation, suggesting that targeting miR-124/VAMP3 may be a potential therapeutic strategy for postoperative diseases involving microglial activation.

#### Cholinergic Anti-inflammatory Pathway in the Pathogenesis of Postoperative Cognitive Dysfunction

Postoperative cognitive decline in elderly mice was associated with the dysfunction of the inflammatory defusing pathway. In recent years, the importance of the cholinergic anti-inflammatory pathway (CAP) in the prevention and treatment of neuroinflammation and cognitive decline caused by sterile trauma has attracted increased attention. Many studies have shown that excessive and persistent cognitive decline and inflammatory responses in elderly mice were associated with CAP dysfunction, and these phenomena could be reversed by α7nAchR agonists (Liu et al., 2019; Gong et al., 2020; Spies et al., 2021). Another study reported that inhalation of sevoflurane impaired the cognitive function of rats, the expression of specific protein 1 (SP1) was significantly increased, and the CAP was inactivated at the same time (Lv et al., 2020). A functional loss detection experiment showed that SP1 gene knockout could rescue the inactivation of CAP and reduce neuroinflammation and apoptosis in the hippocampus of rats, which may provide a new perspective for the treatment of POCD.

#### NMDAR/Ca^2+^/Calpain in the Pathogenesis of Postoperative Cognitive Dysfunction

Previous studies have demonstrated that decreased regulation of BDNF was involved in the pathogenesis of POCD (Qiu et al., 2016a), and accumulating evidence established that dysregulation of BDNF/TrkB signaling contributed to many neurodegenerative diseases (Plotkin et al., 2014; Jerónimo-Santos et al., 2015). Under normal circumstances, BDNF and TrkB-FL combine to conduct signal transduction; however, truncated isoforms of TrkB receptors (TrkB-TC) acted as negative modulators of TrkB-FL receptors (Dorsey et al., 2002; Carim-Todd et al., 2009), and alterations in TrkB-TC:TrkB-FL ratio were thought to trigger and/or reflect dysregulation of BDNF/TrkB signaling (Gomes et al., 2012; Vidaurre et al., 2012). Moreover, in an *in vitro* study, excitotoxic stimulation of cultured rat hippocampal neurons accompanied with glutamate downregulated TrkB-FL while upregulating TrkB-TC receptors, which finally resulted in dysregulation of the BDNF/TrkB signaling (Gomes et al., 2012). This further confirmed the potential correlation. It is well known that calpains are intracellular Ca^2+^-dependent cysteine proteases that play a mechanistic role by cleavage of several substrates, including the TrkB (Jerónimo-Santos et al., 2015), cytoskeletal proteins, and membrane receptors (Gladding et al., 2012). Overactivation of calpains contributed to changes in hippocampal structure, function (Andres et al., 2013), and was even associated with neuronal death (Sugiyama et al., 2017). Furthermore, a calpain-dependent truncated form of TrkB-FL has been found to participate in some neurodegenerative diseases (Jerónimo-Santos et al., 2015; Danelon et al., 2016). When intracellular calcium concentration was increased excessively, there was a corresponding overactivation of calpains, and an important source of increased intracellular calcium concentration was associated with N-methyl-D-aspartate-receptor (NMDAR). NMDARs are ligand-gated ion channels, the dysfunction of which have a strong correlation with neuroinflammation, eventually resulting in deficits of synaptic plasticity and cognitive impairments (Ma et al., 2014; Kumar et al., 2018; Tsoka et al., 2019). Based on these points of view, we hypothesized a potential mechanism: anesthesia and surgery induced neuroinflammation and overactivated NMDARs while the dysfunction of NMDARs triggered the overactivation of calpain leading to the truncation of TrkB-FL, BDNF/TrkB signaling dysregulation, and finally, cognitive impairments. Moreover, a recent study has reported that dysregulation of BDNF/TrkB signaling mediated by NMDAR/Ca^2+^/calpain contributed to postoperative cognitive dysfunction in aging mice while inhibition of NMDAR or calpain by memantine or MDL-28170 treatment reversed the BDNF/TrkB signaling disruption and attenuated cognitive impairments after anesthesia and surgery. This presented a new potential therapeutic strategy for POCD (Qiu et al., 2020).

#### Role of CX3CR1/L1 Signaling in Postoperative Cognitive Dysfunction

Studies have showed that surgical trauma and its accompanying persistent pain could induce the release of systemic inflammation mediators, which led to the activation of microglia to further produce the release of other cytokines and inflammatory mediators, ultimately resulting in cognitive impairment (Cibelli et al., 2010; Plotkin et al., 2014; Jerónimo-Santos et al., 2015; Tejeda et al., 2016). It has been reported that cytokines and chemokines were important mediators of inflammation and inflammatory diseases (Shachar and Karin, 2013; Turner et al., 2014). Moreover, recent studies have shown that the response regulation of cytokines and chemokines in the hippocampus caused by postoperative systemic inflammation and pain was the key factor of cognitive impairment (Terrando et al., 2010; Zhang et al., 2016; Skvarc et al., 2018). Chemokines and other pain mediators regulated the interplay between glial cells and neurons in neuroinflammation and pain conditions, which was critical for maintaining CNS homeostasis (Old et al., 2015). The neuronal chemokine fractalkine (CX3CL1), is expressed in neurons in the brain, while its receptor CX3CLR1 is expressed in microglia. The interaction between the two mediated the activation of glia in the CNS and was vital for many homeostatic processes (Auffray et al., 2007; Ishida et al., 2008; Landsman et al., 2009; Clark and Malcangio, 2014). This raised the question of what the specific mechanism of the CX3CL/R1 is in the signaling pathway affecting cognitive function. A new study showed that transiently elevated CX3CR1 induced persistent pain and increased expression of proinflammatory cytokines after surgery, which consequently contributed to astrocyte activation and increased GABA expression, eventually resulting in cognitive dysfunction. Furthermore, this study revealed the neutralizing antibody blockade of CX3CR1/L1 signaling prevented POCD by reducing proinflammatory cytokine expression. The neutralizing antibody blockade of CX3CR1/L1 also controlled GABA levels in the hippocampus by regulating astrocyte activation (Cho et al., 2021). This provided us with a new perspective that inhibition of CX3CR1/L1 signaling could be a potential target for therapeutic strategies to prevent the development of POCD.

## Conclusion

Although many animal experiments and clinical studies have focused on the role of central nervous inflammation in the occurrence and development of POCD, the specific mechanism has not yet been clarified. In addition, we have made a summary of the non-inflammatory mechanism of POCD in animal experiments in recent years, so as to show the pathogenic mechanism of POCD more comprehensively (Table 2). Many of these trials were limited because they were conducted in animal models; therefore, the relevance of the treatments and the final results in humans are unknown, along with safety and effective dosing information. However, it can be postulated that research on the mechanism of CNS inflammation in the occurrence and development of POCD is likely to play a key guiding role in the prevention and treatment of POCD in clinical practice and lay the theoretical foundation for the search of appropriate anti-inflammatory drugs or anesthesia management programs. POCD occurrence and development are the results of the comprehensive activity of multiple factors during the perioperative period, and the pathogenesis of the condition is multifactorial. The occurrence and development of POCD cannot be fully explained by a single inflammatory response theory. For the clinical treatment of POCD in elderly patients, prevention should be the priority, minimizing the risk factors that may lead to POCD in patients during the perioperative period. Secondly, targeted treatment measures can be applied from the perspective of neuroinflammation, such as the application of anti-inflammatory drugs to reduce the inflammatory response and improve cognitive function. In addition to the application of drugs, functional behavior exercises by patients after surgery and humanistic care from family members are of vital importance. For relevant studies on the pathogenesis of POCD, researchers should further explore the main mechanism of action for neuroinflammation and simultaneously relate it to other possible activity. What is needed is to build a bridge between clinical medicine and basic medicine, from micro to macro, from gene phenotype to biological individual, so that basic medicine can become a proponent for the development and progression of clinical medicine and provide important and practical references as well as guidance for clinical work.

**TABLE 2:** A summary of underlying mechanisms of POCD other than neuroinflammation in animal studies in recent years.

Study focus	Main effects found	References
1. Oxidative stress	Oxidative stress ↑→ POCD↑	Netto et al., 2018; Liu et al., 2019; Ho et al., 2020
2. Mitochondrial function	Mitochondrial dysfunction→POCD↑	Netto et al., 2018; Chen et al., 2020; Wang et al., 2022
3. Synaptic function	Synaptic dysfunction→POCD↑	Zhang X. et al., 2013; Xiao et al., 2018; Gao et al., 2021
4. Neurotrophic support	BDNF↓→POCD↑	Zhang et al., 2014; Fan et al., 2016; Yin et al., 2022
5. Neurodegeneration	(1) Chronic cerebral hypoperfusion→neuronal death→POCD↑ (2) Cerebrospinal Fluid Biomarker for Alzheimer’s Disease contributes to Predicting POCD	Evered et al., 2016; Yamamoto et al., 2018
6. BBB permeability	BBB permeability↑→POCD↑	Wang B. et al., 2017; Cao et al., 2018; Ni et al., 2018
7. Gut-brain axis	(1) Gut microbiome →BBB permeability↑→POCD↓ (2) Unfavorable alterations in gut microbiota and fecal metabolites→POCD↑	Yang et al., 2018; Wen et al., 2020; Lian et al., 2021
8. Epigenetic regulation	(1) Inaction of METTL3→perturbation in m6A RNA methylation signals (2) Histone deacetylases may contribute cognitive impairment (3) Preoperative environment enrichment→preserved neuroligin 1 expression→POCD↓	Luo et al., 2020; He and Wang, 2021; Min et al., 2021
9. Amyloid beta	Amyloid beta↑→POCD↑	Zhang et al., 2020; Kim et al., 2021
10. Tau protein	Tau acetylation↑→POCD↑	Yan et al., 2020
10. NMDAR/Ca^2+^/calpain	NMDAR/Ca^2+^/calpain signal↑→BDNF/TrkB↓→POCD↑	Qiu et al., 2020
11. CX3CR1/L1	CX3CR/L1→astrocyte activation↑→GABA↑ and proinflammatory cytokine expression↑→POCD↑	Cho et al., 2021
12. VEGF	VEGF overexpression→POCD↑	Cao et al., 2019
13. A1A receptor	Activation of the A1A receptor→POCD↑	Zuo et al., 2018
14. Obesity	High-fat diet →Obesity→SIRT1/PGC-1α/FNDC5/BDNF pathway↓→POCD↑	Zhao et al., 2020
15. Iron and glucose metabolism	Cross-dysfunction of iron and glucose metabolism→POCD↑	Ge et al., 2021
16. CeGC	CeGC degradation→BBB permeability↑→POCD↑	Stoddart et al., 2022

*BBB, blood–brain barrier; VEGF, vascular endothelial growth factor; A1A receptor, A1 adenosine receptor; CeGC, cerebral microvascular endothelial glycocalyx. The table above summarizes the results of recent animal studies on the possible pathogenesis of POCD (excluding neuroinflammatory mechanisms). First of all, we must understand that the content of this table is not sufficient and rigorous, and can only be used as a general reference. The table mainly includes two parts, one is the more mature classical pathogenic mechanism, and the other is relatively new and controversial arguments. In addition, through the table content and the review of a large number of related literature, it can be found that the relationship between the various underlying mechanisms of POCD is intricate and difficult to be completely independent. It is likely that one mechanism will play an important role in some part of the other. Furthermore, according to numerous preclinical and clinical studies, neuroinflammation is involved in the pathophysiological process of most of the pathogenic mechanisms of POCD and plays a key role in triggering or maintaining it. For this reason, neuroinflammation has become a research hotspot in recent years.*

## Author Contributions

ZL and YZ prepared the manuscript. YK and SQ drew the figure and tables. JC reviewed and finalized the manuscript, and contributed to funding acquisition. ZL contributed to writing-original draft and visualization. SQ, YK, and JC contributed to writing-review and editing. ZL, SQ, YK, and JC contributed to validation and resources. All authors had full access to all the data in this review and took responsibility for the integrity of the data and accuracy of the data analysis.

## Conflict of Interest

The authors declare that the research was conducted in the absence of any commercial or financial relationships that could be construed as a potential conflict of interest.

## Publisher’s Note

All claims expressed in this article are solely those of the authors and do not necessarily represent those of their affiliated organizations, or those of the publisher, the editors and the reviewers. Any product that may be evaluated in this article, or claim that may be made by its manufacturer, is not guaranteed or endorsed by the publisher.
